# Incidence of oncogenic HPV and HPV-related dysplasia five years after a negative HPV test by self-sampling in elderly women

**DOI:** 10.1186/s13027-022-00453-z

**Published:** 2022-08-03

**Authors:** Ruth S. Hermansson, Matts Olovsson, Inger Gustavsson, Ulf Gyllensten, Olga Lindkvist, Julia Hedlund Lindberg, Gabriella Lillsunde-Larsson, Annika K. Lindström

**Affiliations:** 1 Department of Women’s and Children’s Health, Uppsala University, Uppsala, Sweden; 2 Department of Oncology, Faculty of Medicine and Health, Örebro University, Örebro, Sweden; 3 Department of Immunology, Genetics, and Pathology, Uppsala University, Uppsala, Sweden; 4 Department of Laboratory Medicine, Faculty of Medicine and Health, Örebro University, Örebro, Sweden; 5 School of Health Sciences, Örebro University, Örebro, Sweden

**Keywords:** Cervix, HPV, Cervical intraepithelial neoplasia, Elderly, Self-sampling

## Abstract

**Purpose:**

Cervical cancer prevention for older women can be challenging since there are no specific guidelines for this group**.** This study aimed to determine the incidence of oncogenic HPV and HPV-related dysplasia in elderly women 5 years after being HPV negative.

**Methods:**

Invited women participated five years earlier in a study where self-sampling for HPV testing was applied, at this time, they were all HPV negative. The women were now, five years later invited to perform self-sampling for HPV testing. Women with a positive result performed a repeat HPV test. Those with a positive repeat HPV test were examined by colposcopy, biopsy and cytology.

**Results:**

Of the 804 invited women, 634 (76.9%) agreed to participate in the study and a self-sampling kit was sent to them. Of these, 99.6% (632/634) sent a sample to the HPV laboratory. The participation rate in each age group was 93.3% at age 65, 74.0% at age 70, 80.7% at age 75 and 64.6% at age 80. Overall 18 women (2.8%, 95% CI 3.2 to 6.0) were HPV positive in the first test and 8 (1.3%, 95% CI 0.6 to 2.6) in the second test. Sampling for the second test was done on average 5.4 months after the first test. Fifty per cent (4/8) of the women with a positive repeat test had dysplasia in histology.

**Conclusion:**

The incidence of HPV in previously HPV-negative elderly women was low. Among women who were HPV positive in a repeat test, there was a high prevalence of low grade dysplasia.

## Introduction

Cervical cancer remains a major health problem worldwide with an estimated 530,000 new cases and 273,000 deaths annually [1, 2]. In Sweden, the incidence and mortality rates have substantially declined due to an organized screening program [3]. However, around 550 Swedish women are diagnosed with cervical cancer each year and approximately 200 women per year die from this malignancy. About 25% of cases occur in women 65 years of age and older [4, 5]. Cervical cancer in women above the age of 65 is usually discovered at advanced stages and the prognosis is poor [6]. A lower FIGO stage at diagnosis is strongly associated with cure, since the cancer is highly treatable, and with long-term survival and good quality of life [7].

The primary cause of invasive cervical cancer and precancerous cervical lesions is a persistent infection with oncogenic types of human papillomavirus (HPV*).* There are two main ways to prevent cervical cancer. Primary prevention with prophylactic (HPV) vaccines has been included in the Swedish vaccination program since 2012. Secondary prevention is based on screening for HPV DNA or RNA and HPV-associated cytological abnormalities [8, 9].

The Swedish cervical screening program entails a 3-year interval between negative screens for women aged 23–50, and 7 years for women aged 51–64. Primary screening for oncogenic HPV has recently been recommended for women aged 30–64, while cytology is the primary screening test for women aged 23–29 [10].

The incidence of cervical cancer is higher among women who have not been invited to screening, or have not accepted the invitation to be screened, with significant morbidity and mortality in the unscreened population [11, 12].

Screening for oncogenic HPV infection is found to be more effective than cytology in reducing the incidence of cervical cancer [9, 17]. Self-collection for HPV testing is one recommended approach for increasing access to cervical cancer screening [13]. Self-collection of cervicovaginal samples at home with a return by mail for HPV testing, is a promising approach that may alleviate clinic-based cervical cancer screening barriers, such as transportation limitations or discomfort with pelvic examinations [14, 15]. Repeated self-sampling for an HPV test is a cost-effective strategy within the framework of an organized screening program [16–18].

Cervical cancer prevention in older women can be especially challenging since there are no specific guidelines. The age-specific risks of cervical pre-cancer and cancer among older women with prevalent HPV infection, is not known. It has been suggested that as women leave the screening program, they should be tested for HPV as an exit test, and that surveillance should be continued for women who are HPV positive, but evidence to support this strategy is limited [19, 20]. Besides, it is not known whether changes in hormonal and immunological factors with age can lead to a reactivation of a latent HPV infection, or reduced likelihood for clearance of a new infection [21–23]. The appropriate age for discontinuation of screening is still uncertain and subject to discussion. Studies are also needed to better understand the significance of a new HPV infection among elderly women.

The aim of this study was to determine the incidence of oncogenic HPV and HPV-related dysplasia five years after a negative HPV test among elderly women*.*

## Methods

This prospective population-based longitudinal descriptive study was conducted in Dalarna.

County, Sweden, between August 2019 and May 2020. The study was approved by the Swedish Ethical Review Authority Dnr: 2019–02,490 and 2020–01,576.

**Fig. 1:**
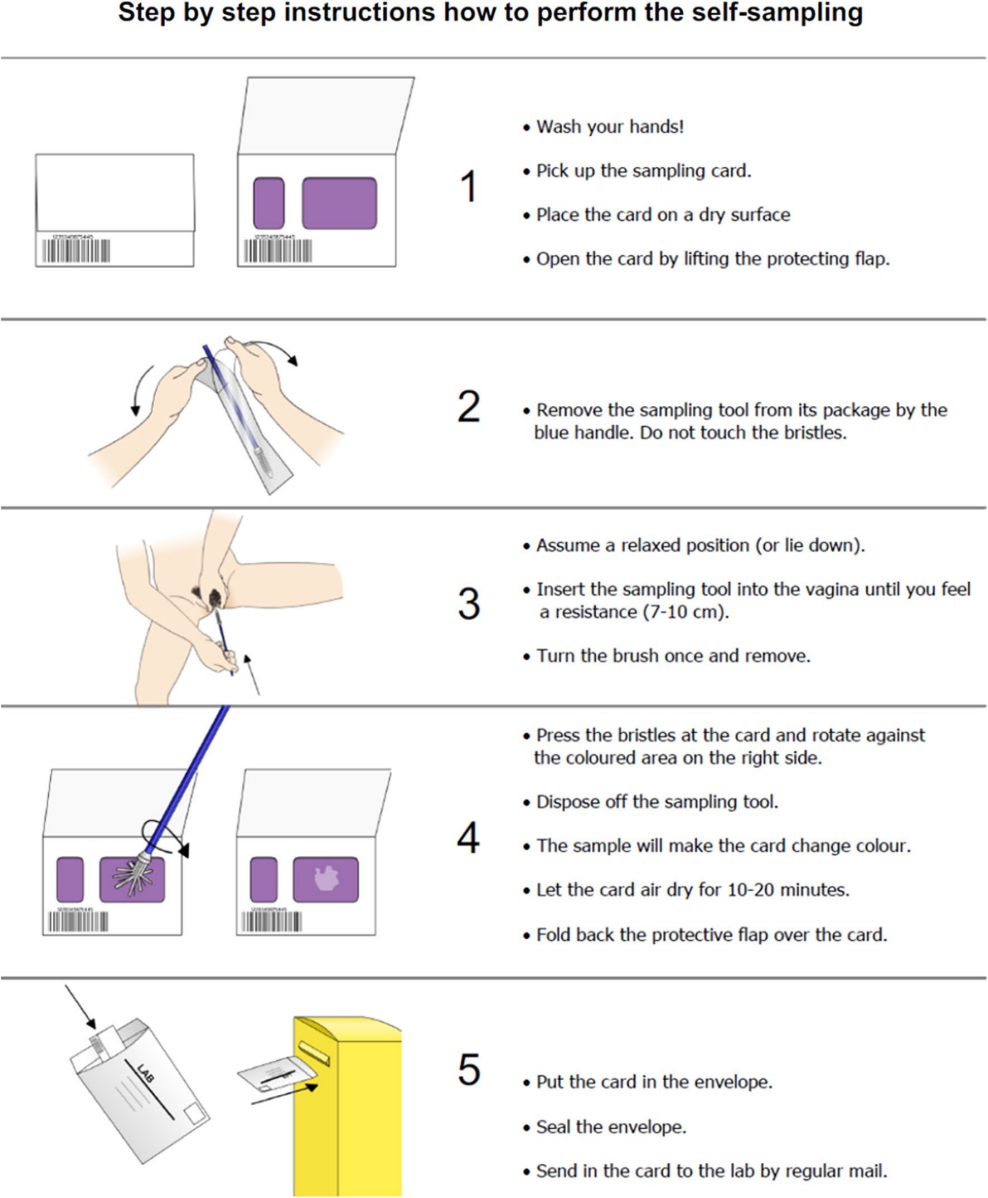
Information supplied to the women on how to perform self-sampling using the Rovers Viba-brush and the FTA card

The study population comprised 804 women between 65 and 80 years old, who five years earlier were randomly recruited and participated in a study on self-sampling for HPV testing. At that time (2014–15) the participants were 60–75 years old and all women were HPV negative [14]. At the time of the first study the screening program ended at the age of 60. The women were now invited to perform a follow-up approximately five years later by self-sampling for HPV testing with the primary aims to determine the incidence of oncogenic HPV and HPV-related dysplasia. Written informed consent was obtained from women who agreed to participate in the study, and self-sampling kits were sent by regular mail. In brief, self-sampling was performed at home (Fig. 1) and the sample was returned, in a prepaid envelope, to the laboratory for analysis of high-risk HPV, as previously described [24]. Women with a positive HPV test were sent a new self-sampling kit four months after the previous test was done. The HPV test was done using a multiplex real-time PCR assay (HPVIR™), as earlier described, which detects the oncogenic HPV types 16, 18, 31, 33, 35, 39, 45, 51, 52, 56, 58, and 59 (18 and 45 are detected together, and 33, 52 and 58 as one group). The sample was collected using the Rovers Viba-brush (Rover Medical Devices B.V., Oss, card GE Healthcare, Cardiff, UK art. no WB129308), and the DNA was extracted from the FTA cards, as described earlier. The threshold for a positive HPV-type was set at ten copies. The HPV typing and distribution of self-sampling kits was undertaken by the HPV laboratory, Department of Immunology, Genetics and Pathology, Uppsala University, Uppsala, Sweden.

Women who were repeatedly positive in the second HPV test were offered examination by colposcopy, sampling for histology, and liquid-based cytology (LBC). Three of the authors (RSH, AL and OL) performed the colposcopies, cervical biopsies, and curettage for histological diagnosis.

Cytotechnicians screened all LBC specimens and those considered abnormal were reviewed by a surgical pathologist. For cervical cytology, the Thin Prep Pap Test was used. The cervical smear was collected with a plastic spatula and a cytobrush. LBC specimens were placed in PreserveCyt solution and processed in the Thin Prep 5000-processor (HologicCytyc Corporation, Boxborough, Mass.). For the classification of cytology and histology, the Bethesda system was used. Specialists in surgical pathology examined the cervical biopsy samples for histologic diagnosis. All cytology and histology specimens were examined at the Department of Pathology and Cytology, Falun County Hospital, Falun, Sweden.

## Results

**Fig. 2:**
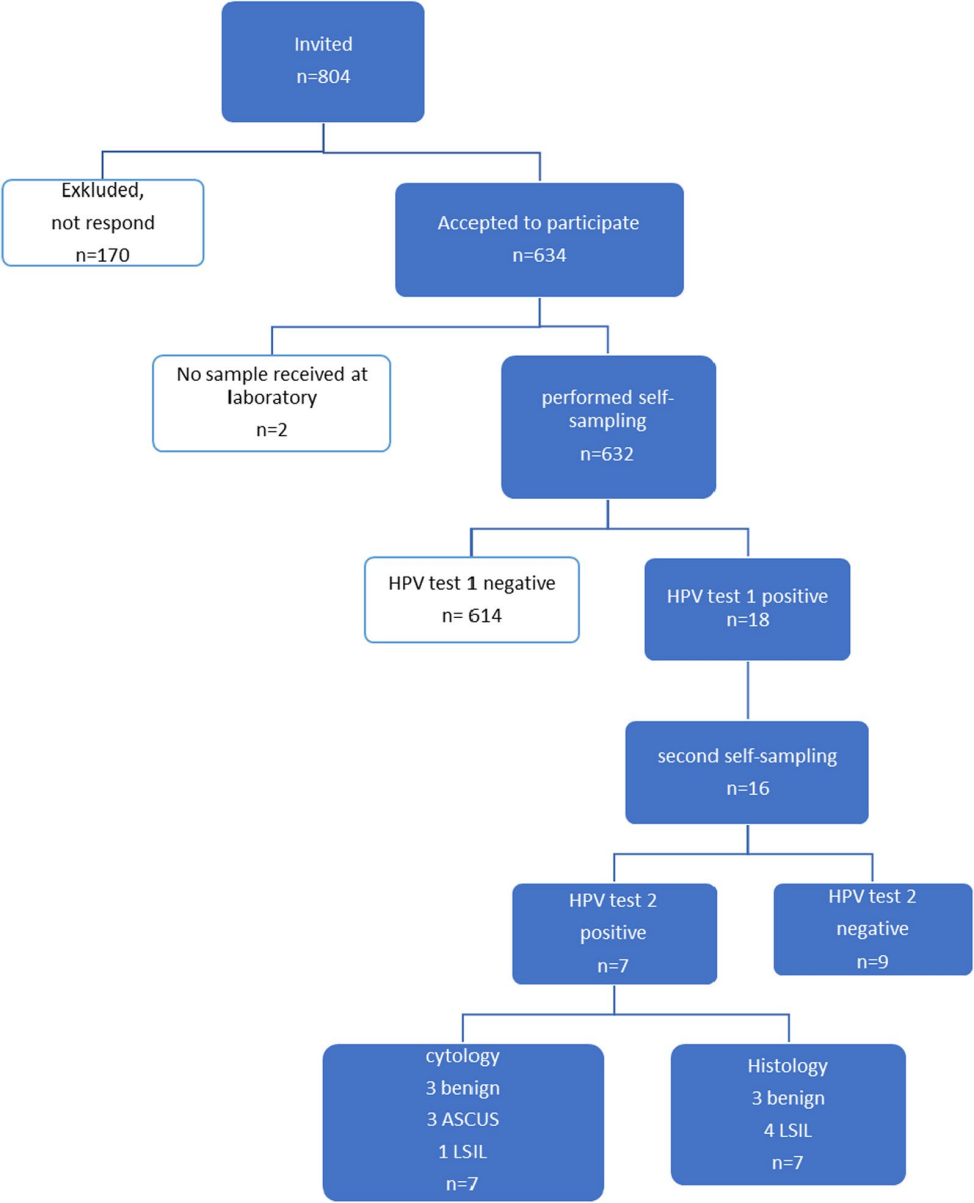
Flowchart showing study design and data on HPV and dysplasia on participants that followed the study protocol. HPV (human papillomavirus), LSIL (Low-grade Squamous cell Intraepithelial Lesion), ASCUS (Atypical Squamous Cells of Uncertain Significance)

Of the 804 invited women, 634 agreed to participate in the study and received a self-sampling kit. Of the 634 participant women, 99.6% (632/634) sent a sample to the HPV laboratory. The participation rate in each age group was 93.3% at age 65, 74.0% at age 70, 80.7% at age 75 and 64.6% at age 80. Four of the women delivered insufficient samples for the HPV assay and those women received a new self-sampling kit for resampling. Samples from all 632 participants were finally analyzed. Overall 18 women (2.8%, 95% CI 3.2 to 6.0) were HPV positive in the first test and 8 (1.3%, 95% CI 0.6 to 2.6) in the second test, collected on average 5.4 months after the first test (Table 1, Fig. 2).

**Table 1:** HPV incidence for the different age groups

Current age, years	HPV negative five years ago n	Self-sampling n (%)		HPV test 1 positive n (%)		HPV test 2 positive n (%)	
65	210	196	(93.3)	7	(3.6)	3	(1.5)
70	219	162	(74.0)	3	(1.9)	2	(1.2)
75	197	159	(80.7)	4	(2.5)	1	(0.6)
80	178	115	(64.6)	4	(3.5)	2	(1.7)
**Total**	**804**	**632**	**(78.6)**	**18**	**(2.8)**	**8**	**(1.3)**

In total 804 women were eligible for and invited to this study and 632 women did send a sample. Of them 18 had a positive first HPV test and the second test ended up with 8 that were still HPV positive

Sixteen women performed a second sampling according to the study protocol. Seven of them had a positive second HPV test and they were examined with colposcopy, sampling for histology and liquid-based cytology (LBC). Since none of the women had a fully visible transformation zone, sampling was performed by cervical abrasion and random biopsies. Results from LBC revealed 3 benign, 3 ASCUS and one CIN1 while colposcopy with cervical abrasion and random biopsies identified 3 benign and 4 LSIL. No one had signs of vaginal dysplasia at the time of colposcopy. Two women performed a second self-sampling after 1.2 and 1.6 months which not was according to the study protocol. One of them had a positive second HPV test. Both had a non-visible transformation zone at colposcopy and both cytology and histology were benign. No glandular atypia was diagnosed. Women who were HPV negative in the second HPV test, were scheduled for a follow up HPV test one year later after the second one. There was no significant difference in HPV incidence between the different age groups (Table 1). HPV 51 was the most prevalent type (Table 2).

**Table 2:** Data on HPV type and results for cytology and histology on samples from elderly women who were HPV positive (n = 18)

Current age	HPV test 1	HPV test 2	Cytology	Histology
70	31	31	Benign	Benign
65	51	51	Benign	Benign
70	51	51	LSIL	LSIL
75	51	51	Benign	LSIL
80	56	56	Benign	Benign
80	18/45	18/45	ASCUS	Benign
65	33/52/58	33/52/58	ASCUS	LSIL
65	33/52/58	33/52/58	ASCUS	LSIL
65	18/45	negative		
65	51	negative		
65	51	negative		
65	33/52/58	negative		
70	18/45	negative		
75	18/45	negative		
75	39	negative		
75	31	negative		
80	16	negative		
80	33/52/58	negative		

*LSIL* low grade squamous intraepithelial lesion; *ASCUS* atypical cells of undetermined significance

## Discussion

The significance of the detection of an HPV infection among earlier HPV-negative older women is not well studied. One previous study reports on the incidence of HPV and HPV-related dysplasia in women who were HPV negative some years earlier and older than 60 years. It was concluded that older women becoming HPV positive were at high risk of dysplasia [25]. Ageing itself is also a risk factor for cancer disease and most chronic diseases [26]. Still, the impact of age on the risk of developing severe dysplasia or cervical cancer among elderly women with a newly detected HPV infection is unknown. An important finding in the current study is that there was no HSIL or cervical cancer among these women, who had a negative HPV test five years earlier. This is in agreement with what is known from younger women, that severe dysplasia and cervical cancer among HPV-negative women is quite rare [27], and that the time from being infected with high-risk HPV until the presence of HSIL and cervical cancer, is more than five years in most cases [28].

Studies on the HPV incidence in elderly women are few, but several earlier studies have shown that the HPV prevalence is highest around age 25 and then decreases with age [20, 29, 30]. If one compares different age groups of women 50 years and older, when most women are postmenopausal, the prevalence seems to be rather similar [20, 29]. In our previous studies on elderly women, we have not been able to show any differences in HPV prevalence between age groups [14, 31]. In this study there also seem to be no differences in HPV incidence between the age groups (Table 1). The possibility to draw firm conclusions on this is however limited since the numbers of cases is very small.

There are controversies over when to stop screening for cervical cancer, and regarding the significance of a negative exit test [31]. Screening practices for postmenopausal women are challenging since both cervical cytology and colposcopies are less accurate among these women. Even if HSIL or cervical cancer were not found in this study, it is impossible to ensure that neither were present, since the transformation zone was not visible during the colposcopy, and biopsies were randomly collected. A recent study showed that a persistent HPV infection needs to be monitored despite benign cytology and colposcopy, since 6/19 (32%) women with a persistent and ongoing HPV infection were revealed to have an undiagnosed CIN2 + when LEEP was performed [32]. The PPV is an indicator of the significance of having a persistent HPV infection for the risk to have dysplasia. In this case, the PPV is probably underestimated since conization probably would have revealed more cases with dysplasia, as has been shown in other studies [32].

In this study, the incidence of HPV five years after a previous negative HPV test was lower than in the general population of elderly women [30, 33], but fifty percent of the women with a positive repeat test had dysplasia in histology. We were unable to determine whether these newly detected infections resulted from a new sexually-acquired HPV infection, a reactivated latent infection acquired many years earlier, or whether the previous test was negative due to a viral load under the threshold for a positive test, i.e. less than ten copies. Thus, the HPV infections detected at the 5-year follow-up may constitute a combination of newly acquired and persistent infections.

It is not known whether a woman's age influences her risk for HSIL or cervical cancer in the context of HPV positivity. The significance of a persistent HPV infection with or without LSIL is not well studied in the older age group. It is evident that young women´s data are not necessarily applicable to postmenopausal women and especially not elderly women. Mechanisms involved might include changes in local immune defense, blood perfusion, tissue atrophy, and vaginal microbiota [21, 26, 34].

There are some potential limitations with the study, and they include the participants coming from one single region of Sweden, which might reduce the generalizability of the obtained results. Another weakness is that there was no data on sexual partners during the last 5 years which could have explained some of the cases with HPV positivity. Finally, the histological data were obtained by, in many cases, randomly collected cervical biopsies, which means that there might have been HSIL or cervical cancer in the invisible part of the cervix, in analogy with what has been seen in the study by Aarnio et al. [32]. In these cases, it might have been advisable to perform diagnostic conization. The strengths of the study were a relatively high participation rate, that the vast majority followed the study protocol, and that the follow-up with a gynecologic examination including cytology and colposcopy with biopsy was performed on all women with a positive second HPV test.

## Conclusion

The incidence of HPV positivity in previously HPV-negative elderly women was low but low grade dysplasia was common in those with repeat positive tests. Additional research is needed to determine whether elderly women with one negative HPV test are sufficiently protected against cervical cancer.

## Data Availability

The datasets generated during and/or analyzed during the current study are available on reasonable request. Authors may be contacted at Uppsala University. Department of Women’s and Children’s Health. Dag Hammarskjölds Väg 14B, 75,237 Uppsala Sweden. kbh@kbh.uu.se.
